# Novel orphan medicines and abandoned pathways - the US Orphan Drug Act of 1983 and the impact on rare rheumatologic diseases and lysosomal storage disorders

**DOI:** 10.1186/2194-7791-2-S1-A1

**Published:** 2015-07-01

**Authors:** Markus Ries, Thomas Lutz, Anette Lampert, William Mountford, Konstantin Mechler, Georg F Hoffmann

**Affiliations:** Pediatric Neurology and Metabolic Medicine, Center for Pediatric and Adolescent Medicine and Center for Rare Disorders, University Hospital Heidelberg, Heidelberg, Germany; Pediatric Rheumatology, Center for Pediatric and Adolescent Medicine and Center for Rare Disorders, University Hospital Heidelberg, Heidelberg, Germany; Clinical Pharmacology and Pharmacoepidemiology, Cooperation Unit Clinical Pharmacy, University Hospital Heidelberg, Heidelberg, Germany; Quintiles, Epidemiology, Wilmington, NC USA; Pediatric Psychopharmacology, Department of Child and Adolescent Psychiatry and Psychotherapy, Central Institute of Mental Health, Medical Faculty Mannheim, University of Heidelberg, Mannheim, Germany

## Aims

We asked the research question, how many orphan drugs were intended to be developed in the area of rare rheumatologic diseases (RRDs) and lysosomal storage disorders (LSDs), measured as the number of orphan drug designations at the FDA between 1983 and 2013. In addition, we analyzed the technology platforms and factors for successful registration.

## Methods

Analysis of the FDA database for orphan drug designations.

## Results

**RRDs** - in the last three decades, out of 77 orphan drug designations, 14 FDA orphan drug approvals were granted for RRDs, i.e. juvenile idiopathic arthritis (N = 5), cryoporin associated periodic syndromes (N = 3), uveitis (N = 3), familial Mediterranean fever (N = 1), anti-neutrophil cytoplasmic antibody-associated vasculitis (N = 1), xerostomia/ keratoconjunctivitis sicca in Sjögren's syndrome (N = 1). Mean time (SD) from designation to approval was 3.9 (2.81) years. 6/14 FDA-approved drugs were small molecules, 8/14 were biologics. 15/77 orphan drug designations were withdrawn. Despite the rarity of conditions, 13/14 pivotal studies were randomized controlled trials.

**LSDs:** Orphan drug status was designated 70 times for 20 conditions. Fourteen drugs for seven conditions received FDA approval. Orphan drug status was designated for enzymes, modified enzymes, fusion proteins, chemical chaperones, small molecules, stem cells and gene therapies. Approved therapies were enzyme replacement (N = 10), substrate reduction (N = 1), and small molecules (N = 3). FDA approval was statistically significantly associated with a disease prevalence ≥ 0.5/100,000 (P =.007) and clinical development programs that did not require a primary neurological endpoint (P < .001).

## Conclusions

During the last three decades, 14/77 orphan drug designations were approved for the treatment of RRDs. Likewise, 14/70 orphan drug designations were approved for LSDs. Pivotal studies were small clinical trials. These data provide an insight into the drug pipeline and abandoned pathways.

